# Decimal systems around the world

**DOI:** 10.1098/rstb.2024.0211

**Published:** 2025-10-20

**Authors:** Ezequiel Koile, Damián Blasi

**Affiliations:** ^1^ Pontifical Catholic University of Peru, San Miguel 15088, Lima, Peru; ^2^ Department of Linguistic and Cultural Evolution, Max-Planck-Institute for Evolutionary Anthropology, 04103 Leipzig, Germany; ^3^ Catalan Institution for Research and Advanced Studies (ICREA), 08010 Barcelona, Spain; ^4^ Center for Brain and Cognition, Pompeu Fabra University, 08002 Barcelona, Spain

**Keywords:** numerals, decimal systems, anchor, transparency, base-10, canonicity, compositionality

## Abstract

Decimal numeral systems (base-10) are the most widespread type of counting system across the world’s languages. Their ubiquity has often been attributed to presumed functional advantages, including ease of use, cognitive efficiency and arithmetical transparency. In principle, decimal systems represent numbers as compositions involving a form representing 10 and smaller atomic numerals (e.g. Mandarin *shí sān* ‘thirteen’, literally ‘ten three’). In practice, however, many languages exhibit deviations that obscure this compositional logic—such as allomorphy (*fifteen* in English, as opposed to *five-ten*), suppletion (e.g. Russian *sorok* ‘forty’, unrelated to *četyre* ‘four’ or *desjat'* ‘ten’), or secondary structures involving other compositional elements (e.g. 5). Despite being frequently mentioned in the literature, these violations have not been systematically analysed at scale. Here, we develop a rule-based classification of decimal systems and apply it to a curated, genealogically and geographically balanced sample of 118 languages. We assess each system along three dimensions—*transparency*, *canonicity* and *compositionality*—and show that, while surface-level irregularities are widespread, the underlying mathematical structure of decimal systems remains highly regular and patterned. These results reveal that deviations from perfect decimality are not random but instead reflect structured variation shaped by historical, cognitive and typological constraints.

This article is part of the theme issue ‘A solid base for scaling up: the structure of numeration systems’.

## 1. Introduction

Decimal numeral systems in the world’s languages are characterized by the reuse of the word for 10 or a variant of it when composing higher numerals.^1^ For instance, Mandarin Chinese and German employ systems of this type, which is apparent in higher numerals such as 17 (*shí qī*, literally ‘ten seven’ in Mandarin, and *sieb-zehn*, literally ‘sev(en) ten’ in German) or 24 (*èr shí sì*, literally ‘two-ten four’ in Mandarin, and *vier-und-zwanzig* , literally ‘four and twenty’ in German^2^). Decimal numeral systems are the most frequently attested across human societies and incidentally the most studied [1–3]. In a large-scale sample of languages, roughly 60% utilize a decimal numeral system [4].

In addition, alternatives to decimal systems seem to be peculiarly clustered regionally. For example, systems whose main compositional element is 20 are frequent in Mesoamerica [5,6] and the Caucasus [7,8,9], and systems whose main compositional element is 2 are frequent in Australia [10], Papunesia [11] and South America [12].^3^ All of this has strengthened the conjecture that decimal numeral systems are not simple accidents of history but that, instead, they are coherent with human preferences that might be communicative [14], cognitive [15–17], anatomical [18] and/or driven by cultural evolution and transmission [19,20].

Some of the purported utility brought by these systems rests upon them being overtly compositional in their form [16]. In other words, it is implicitly understood that a decimal system is defined by a specific set of rules constraining the form of its numerals, based on the re-utilization of the word for 10 in higher numerals.^4^ However, these rules are often flouted in natural languages. The presence of allomorphs (different realizations of a morpheme with a unique meaning), as well as exceptions in the rules, lowers their transparency (see table 1). However, linguistic typology (the discipline that categorizes and systematizes linguistic patterns in the world’s languages) classifies languages using ideal types. In practice, such irregularities are weighed against the overall structure when classifying a system as decimal.

**Table 1.** Most frequent violations of ideal decimal types. Curly brackets stand for allomorphic forms and square brackets for suppletions.

language	language family	area	rule violation	component	number	numeral	gloss
Mandarin Chinese	Sino-Tibetan	Asia	none	—	23	èr shí sān	two-ten three
Italian	Indo-European	Western Europe	allomorphy	base	13	ˈtredit͡ʃi	three {ten}
Corsican	Indo-European	Western Europe	allomorphy	atom	15	ˈkwindɛd͡ʒi	{five} ten
English	Indo-European	global	allomorphy	base and atom	13	ˌθɜːˈtiːn	{three} {ten}
Seneca	Iroquoian	North America	suppletion	base	13	së́ sɡaːeʼ	three [ten]
Telugu	Dravidian	Asia	suppletion	atom	15	pədəhenu	{ten} [five]
Oriya	Indo-European	Asia	suppletion	base and atom	16	ʃohɭɑ	[sixteen]
Didinga	Surmic	Africa	quinary substructure	atom	17	ɔmɔt̪ɔ xɪ́ t̪ʊ́ɾkɪ́ɾámːá	ten five-two
Kunama	isolated	Africa	quinary substructure	base	80	ʃěbkōntásàttê	ten five-three

In an ideal decimal numeral system, a composed numeral is formed either by addition or multiplication (or both) between the word form representing the value 10 and at least one other lower numeral. The numerals representing values lower than 10, which themselves do not have an inner structure, are called *atoms*, while 10 is the *base*, meaning intuitively ‘[a] numerical value to which an arithmetic operation is applied so as to form a numeral’ [21].^5^ A stronger definition requires a regularity in the use of one or more arithmetic operations, typically addition and multiplication in the case of linguistic numeral systems (*cycle base* in terms of [21]). An example of a perfectly decimal system, at least for the range 1−100, is Mandarin Chinese (see table 1). Violations of perfect decimality can be apparent in either the form representing the base-10 or the form of the atomic numeral acting as an addend or multiplier to the base-10. Table 1 shows examples of all these combinations present in languages of the world.^6^

The variation shown in table 1 illustrates that over-arching claims about the utility, function and the presence of decimal numeral systems (as well as other bases) are conditioned by the frequency and the nature of potential violations of these rules [16,17]. However, these violations have not yet been the object of systematic and comprehensive analyses. Here, we provide a succinct and informative study of the way in which decimal numeral systems comply with or defy their ideal rules of compositionality. We gather and curate a balanced sample of these systems comprising *n* = 118 languages of 110 different language families and all the major world regions where these systems have been attested. We evaluate the rules involved in the construction of higher numerals, and we estimate the compliance of individual numerals covering the additive cycles 11−19 and 21−29, as well as the multiplicative cycle 20−90, distinguishing between the rules from the point of view of the base (i.e. the numeral representing 10) and the addends or multipliers accompanying it. We then extract the most commonly occurring patterns of rule violations and draw conclusions for the study of numeral systems in general.

## 2. Data and methods

### (a) Data

We use a genealogically and geographically balanced sample of decimal numeral systems taken from Numeralbank, the largest cross-linguistic database on numeral systems to date [4]. Numeralbank contains word forms for numerals from over 5000 languages of the world. Sixty per cent of the entries for these languages include a ‘complete set’ of numerals, defined here as all numerals between 1 and 30 plus the multiples of ten from 40 to 100.^7^ Non-complete sets are the result of either an absence of one or more of those numerals in a given system or a simple lack of data in its description. Eighty per cent of all the numeral systems are annotated for base, although the criteria for this definition vary (see electronic supplementary material). We have access to 2577 languages with both base annotation and a complete set of numerals. Of these, 1712 (66%) are annotated as decimal (base-10), 465 (18%) as quinary (base-5), 326 (13%) as vigesimal (base-20) and 74 (3%) as other.^8^ In addition, for most of these languages, we have a manually annotated mathematical gloss describing the underlying composition of each numeral. For example, English 13 *thirteen*, although not containing the exact forms *three* or *ten,* is mathematically glossed as ‘3 + 10’ owing to its clearly decimal structure.

**Figure 1.**
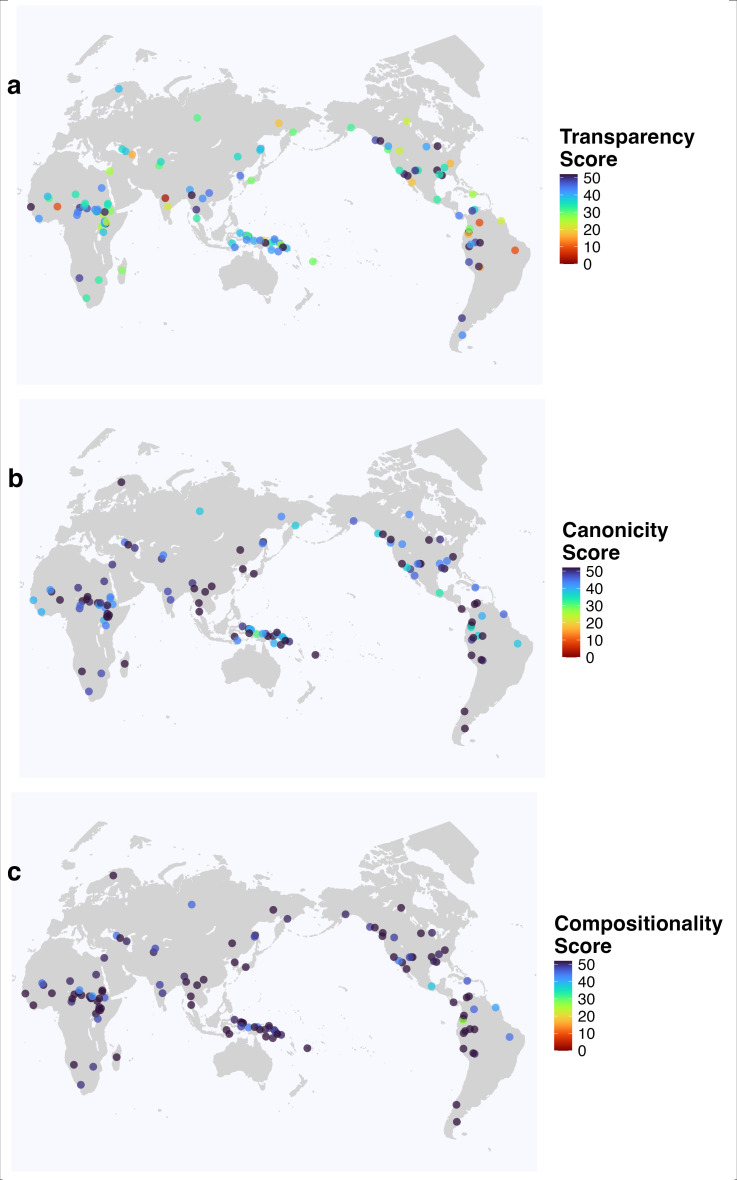
Total number of perfect decimal rules satisfied by each decimal system in our sample. (a) The transparency score (how much the forms for complex numerals perfectly reuse lower numerals), (b) the canonicity score (how perfectly decimal their underlying structure is) and (c) the compositionality score (how frequently the gloss for a lower numeral is reused in a larger numeral).

Based on the six linguistic macro-areas typically used in language comparison, as well as the language family to which the language belongs, we randomly sampled one group from each area and family [22]. More concretely, if one family is completely contained in one geographical macro-area, we sampled only one language from that family, while if it contains member languages in multiple macro-areas, we picked one language in each macro-area. After this, we manually curated the sample, making sure that each system indeed shows at least a superficial decimal structure, and if this was not the case, we replaced it with another in the same family and macro-area. This procedure yielded a sample of 118 languages, displayed in figure 1 (see also electronic supplementary material, figure S1).

## 3. Methods

We define a set of explicit rules to capture the compositionality of decimal numeral systems. These are local rules that apply to individual numerals. Specifically, we consider the implicit compositional structure in each numeral as


(3.1) Z=X⧫Y,

where ♦ stands for either addition (+) or multiplication (×).^9^ Each of these rules can be interpreted in three different ways depending on the question of interest.

For studying *transparency* of numeral systems, we ask whether the *word form* for a complex (higher) numeral contains the word form for one or more simpler (lower) numerals. For studying *canonicity*,^10^ we ask whether a complex (higher) numeral is ‘perfectly decimal’, meaning whether it is formed by atomic numerals following the canonical rules of a decimal base. Finally, for studying *compositionality,* we ask whether the underlying mathematical glossing of a complex numeral contains that of one or more simpler numerals.

More concretely, for the transparency study, we jointly consider the following rules deriving from equation (3.1):

‘Is the word form for *X* included in the word form for *Z*?’           (rule 1)

‘Is the word form for *Y* included in the word form for *Z*?’           (rule 2)

For the canonicity study, we test the homologous rules:

‘Is the exact mathematical value of *X* included in the glossing of *Z*?’     (rule 1′)

‘Is the exact mathematical value of *Y* included in the glossing of *Z*?’     (rule 2′)

For the compositionality study, we test the homologous rules:

‘Is the glossing of *X* included in the glossing of *Z*?’              (rule 1′′)

‘Is the glossing of *Y* included in the glossing of *Z*?’              (rule 2′′)

We evaluate a total of 52 rules for each study (transparency, canonicity and compositionality) for each decimal system, divided into six groups (i)–(vi). These are as follows:

In the transparency study:

For teens (11–19):

Expected form: *Z* = 10 + *Y*.

(i) ‘Is the word form for 10 included in the word form for *Z*?’ (*Z* = 11–19)
(ii) ‘Is the word form for *Y* included in the word form for *Z*?’ (*Z* = 11–19, *Y* = 1–9)

For twenties (21–29):^11^

Expected form: *Z* = 20 + *Y*.

(iii) ‘Is the word form for 20 included in the word form for *Z*?’ (*Z* = 21–29)
(iv) ‘Is the word form for *Y* included in the word form for *Z*?’ (*Z* = 21–29, *Y* = 1–9)

For decades (20–90):

Expected form: *Z* = 10 × *Y*.

(v) ‘Is the word form for 10 included in the word form for *Z*?’ (*Z* = 20–90)
(vi) ‘Is the word form for *Y* included in the word form for *Z*?’ (*Z* = 20–90, *Y* = 2–9)

In the canonicity study:

For teens (11–19):

Expected form: *Z* = 10 + *Y*.

(i′) ‘Is the exact mathematical value of 10 included in the glossing of *Z*?’ (*Z* = 11–19)
(ii′) ‘Is the exact mathematical value of *Y* included in the glossing of *Z*?’ (*Z* = 11–19, *Y* = 1–9)

For twenties (21–29):

Expected form: *Z* = 20 + *Y*.

(iii′) ‘Is the exact mathematical value of 20 included in the glossing of *Z*?’ (*Z* = 21–29)
(iv′) ‘Is the exact mathematical value of *Y* included in the glossing of *Z*?’ (*Z* = 21–29, *Y* = 1–9)

For decades (20-90):

Expected form: *Z* = 10 × *Y*.

(v′) ‘Is the exact mathematical value of 10 included in the glossing of *Z*?’ (*Z* = 20–90)
(vi′) ‘Is the exact mathematical value of *Y* included in the glossing of *Z*?’ (*Z* = 20–90, *Y* = 2–9)

And in the compositionality study:

For teens (11–19):

Expected form: *Z* = 10 + *Y*.

(i″) ‘Is the glossing of 10 included in the glossing of *Z*?’ (*Z* = 11–19)
(ii″) ‘Is the glossing of *Y* included in the glossing of *Z*?’ (*Z* = 11–19, *Y* = 1–9)

For twenties (21–29):

Expected form: *Z* = 20 + *Y*.

(iii″) ‘Is the glossing of 20 included in the glossing of *Z*?’ (*Z* = 21–29)
(iv″) ‘Is the glossing of *Y* included in the glossing of *Z*?’ (*Z* = 21–29, *Y* = 1–9)

For decades (20–90):

Expected form: *Z* = 10 × *Y*.

(v″) ‘Is the glossing of 10 included in the glossing of *Z*?’ (Z = 20–90)
(vi″) ‘Is the glossing of *Y* included in the glossing of *Z*?’ (*Z* = 20–90, *Y* = 2–9)

For example, if we apply these rules to English 13 *thirteen*, the rule of the set (ii) for transparency ‘Is the word for 3 included in the word for 13?’ is not satisfied (since *three* is not included in *thir-teen*), while the equivalent rule from the sets (ii′) ‘Is the exact mathematical value of 3 included in the glossing of 13?’ and (ii″) ‘Is the glossing of 3 included in the glossing of 13?’ are satisfied. Conversely, for Didinga (Surmic) 17 *ɔmɔt̪ɔ xɪ́ t̪ʊ́ɾkɪ́ɾámːá* (literally ‘ten five-two’), the rule of set (ii) ‘Is the word for 7 included in the word for 17?’ is satisfied, the rule (ii″) ‘Is the glossing of 7 included in the glossing of 17’ is also satisfied, since 7 *t̪ʊ́ɾkɪ́ɾámːá* is included in the form for 17, but the rule (ii′) ‘Is the exact mathematical value of 7 included in the glossing of 17?’ is not, as the glossing is ‘10 + 5 + 2’ instead of ‘10 + 7’ with an atomic form for 7 (see more details in §4).

We tested each of these 52 rules for each decimal system in our sample at all three levels presented and characterized these systems according to their level of fulfilment.

## 4. Results

An overview of the results is shown in figure 1, which displays the cumulative number of rules satisfied by each system; this information is visualized in the map in figure 1a for transparency, in figure 1b for canonicity, and in figure 1c for compositionality.

From the transparency study (figure 1a), we found 12 ‘perfectly transparent’ decimal systems, satisfying all 52 rules, and one system with as few as only three rules satisfied, while most systems display intermediate values. We can observe a moderate geographical bias, as lower transparency occurs mostly in languages of India and eastern South America, while systems in Africa and Papunesia are highly transparent on average. For the canonicity analysis (figure 1b), we find that 52 languages satisfy all 52 rules, while the lowest number of rules satisfied is 28. We observe higher scores overall, as expected since these rules are easier to satisfy, and a more balanced distribution, apart from lower values in Papunesia (see below). Finally, for the compositionality study (figure 1c), we observe 76 languages (64% of the sample) satisfying all 52 rules, while the lowest number of rules satisfied is 38, which is almost three-quarters of all rules.

**Figure 2.**
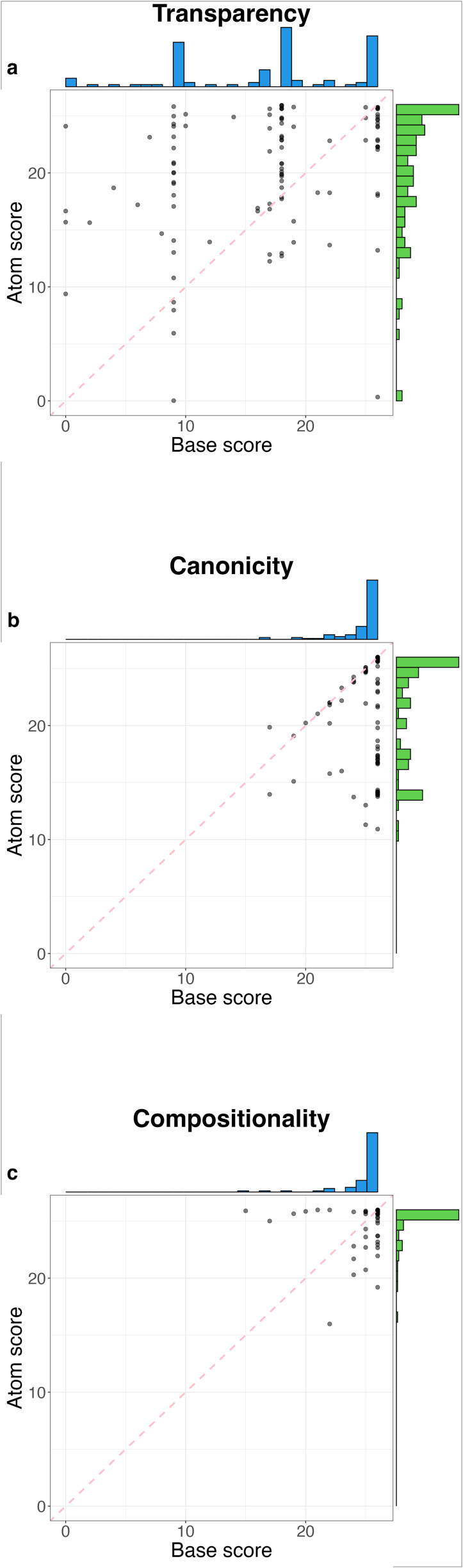
Aggregated rules for the base versus aggregated rules for atoms for each individual language in the sample for (a) transparency, (b) canonicity and (c) compositionality.

We now disaggregate the total number of rules for each numeral system into two groups of 26 rules each: one for those involving the transparency/canonicity/compositionality of the base ((i), (iii), (v); (i′), (iii′), (v′); and (i″), (iii″), (v″), respectively, from §3) and those involving the remaining atomic numerals (rules (ii), (iv), (vi); (ii′), (iv′), (vi′); and (ii″), (iv″), (vi″)). Each of these scores runs from 1 to 26. Results are shown in figure 2a for transparency, figure 2b for canonicity and figure 2c for compositionality.

**Figure 3.**
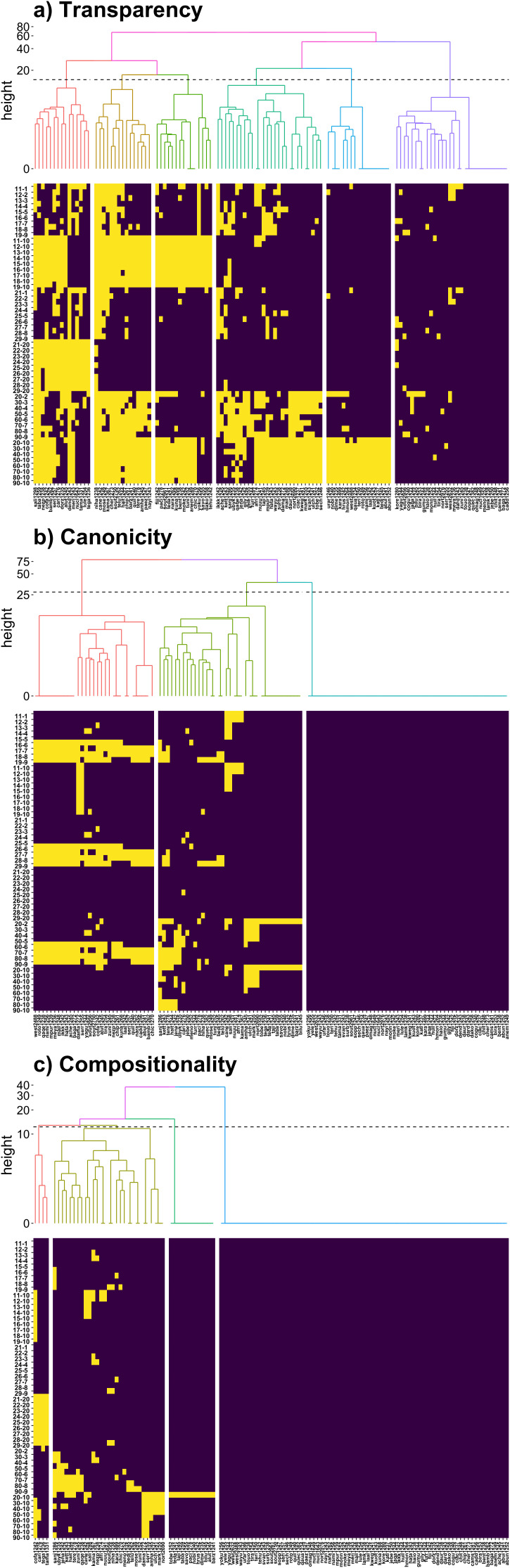
Lower part of each panel: distribution of rules (*y*-axis) satisfied (blue) and not satisfied (yellow) in each numeral system in the sample (*x*-axis). Upper part of each panel: dendrogram for clustering of these numeral systems into types for each analysis (‘height’ stands for dissimilarity between numeral systems and clusters of them, plotted in a logarithmic scale). (a) Transparency: six clusters are found, each of them satisfying or not each set of rules (i)–(vi) (almost) entirely. (b) Canonicity: three clusters, representing perfectly decimal systems, almost perfectly decimal, and mostly quinary-decimal mixed systems. (c) Compositionality: four clusters with (perfectly) decimal behaviour, non-decimality for the twenties and other violations of the rules, especially on the decades.

In the transparency analysis (figure 2a), we observe two very different behaviours: while words for atoms tend to be expressed transparently as part of complex numerals (both additively and multiplicatively, histogram in green), the words for the base appear explicitly only in pockets—out of all the rules of this group, most systems satisfy either none, one-third, two-thirds or all of them (histogram in blue), clearly reflecting the different sets of rules (i), (iii) and (v). This behaviour is shown in further detail in figure 3. In addition, the scatter plot in figure 2a shows a hierarchy in transparency among the two groups, as it is necessary for a high number of atom rules ((ii), (iv) and (vi)) to be satisfied in order to have base rules ((i), (iii) and (v)) satisfied too. In other words, there are almost no cases in which all transparency rules involving the base are satisfied but the atomic rules are not, while the opposite is not true: there are cases with perfectly transparent atoms, but a non-transparent base.

In the study of canonicity (figure 2b), a different behaviour is present. Rules for the base are overwhelmingly satisfied (histogram in blue), while those for atoms, although mostly satisfied, tend to have a small number of irregularities (histogram in green). This behaviour can be explained by the existence of non-decimal, smaller compositional elements, which we will refer to as ‘anchors’ [13,21]. In particular, a quinary structure (anchor-5) is present in several cases [23]. An example of this is Didinga (Surmic), where numerals for 6 through 9 make use of the numeral for 5 in their formation (e.g. 7 *t̪ʊ́ɾkɪ́ɾámːá* is composed of 5 *t̺úɾ* and 2 *ràmːá*).^12^ Therefore, composed forms reusing these non-atomic lower numerals are not perfectly decimal (e.g. 17 *ɔmɔt̪ɔ xɪ́ t̪ʊ́ɾkɪ́ɾámːá* is composed of 10 *ɔmɔt̪ɔ,* 5 *t̺úɾ* and 2 *ràmːá*). Consequently, the full gloss for 17 is ‘10 + 5 + 2’. Although 10 is a constituent part of this numeral, and so the base rule ‘17 = 10 + *Y*’ is satisfied, 7 as an atom is not, and so the atom rule is not satisfied, since ‘17 = *X* + 7’ is not true in the glossing. This is one example where the canonicity rule is not satisfied, while the transparency rule is (since 7 *t̪ʊ́ɾkɪ́ɾámːá* is indeed a constituent part of 17 *ɔmɔt̪ɔ xɪ́ t̪ʊ́ɾkɪ́ɾámːá* , regardless of its non-atomic structure). In this case, the compositionality rule is satisfied as well, since ‘5 + 2’ is the gloss for 7, which is part of the gloss of 17.

Finally, in the study of compositionality (figure 2c), most rules are satisfied, with very few exceptions, both in the case of atom rules and in the case of base rules. This is due to the inherent flexibility of these rules, which are expected to be satisfied by definition in any system classified as decimal. Details of the violations of these rules are given in figure 3.

**Figure 4.**
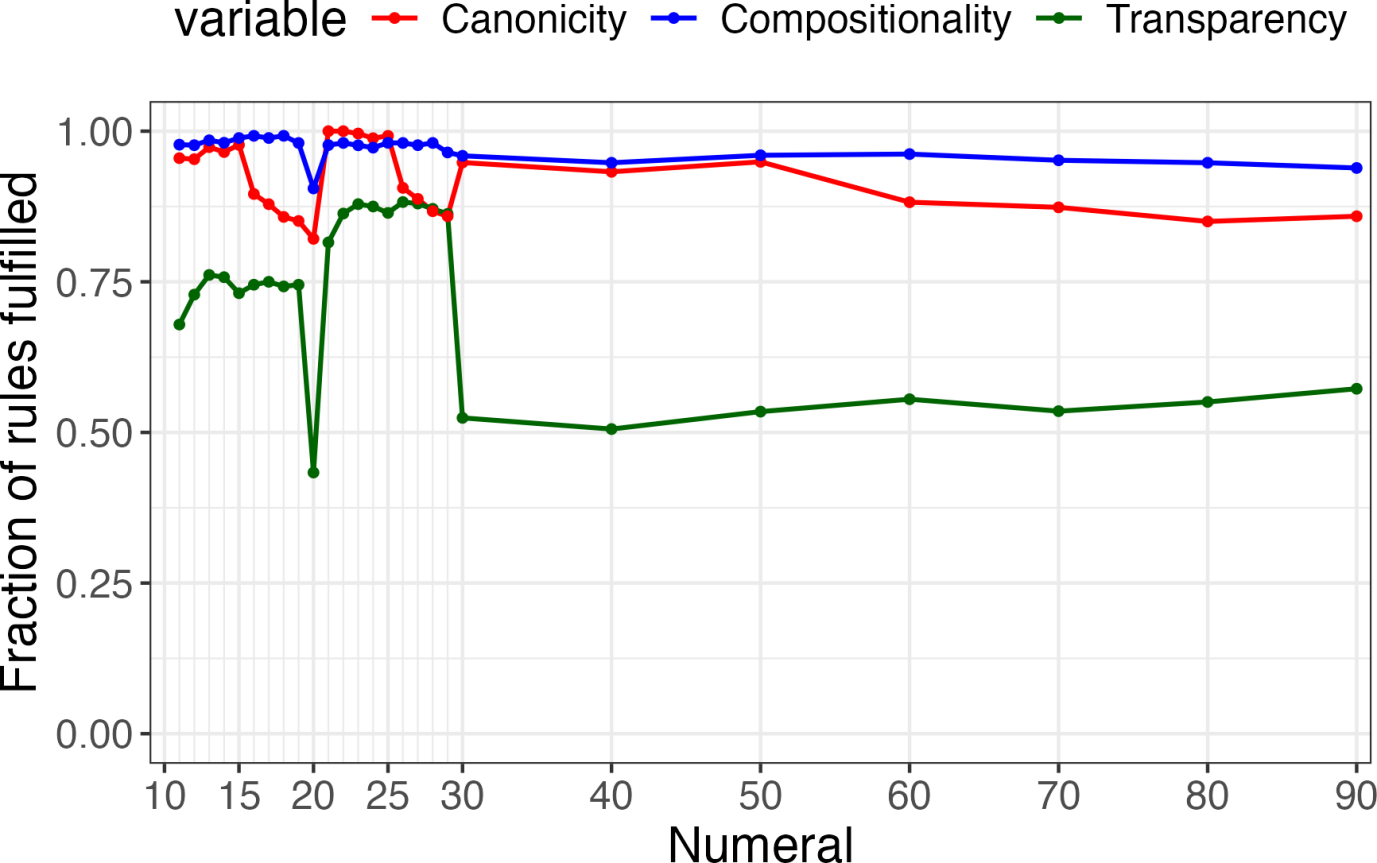
Aggregated score for transparency, canonicity and compositionality across the global sample of decimal systems for each individual numeral.

Figure 4 provides a summary of the degree to which complex numerals in decimal systems are transparent, canonical and compositional. We plot the aggregated score of rules for each numeral system in our sample, ranging from a normalized score of 0 (none of the rules is satisfied in any of the languages of the sample) to 1 (all 52 rules are satisfied). We observe the following patterns: (i) Compositionality is practically always satisfied, followed by canonicity and transparency, as expected. (ii) Decades (multiples of 10) tend to be less transparent than interdecadal numerals (11–19 and 21−29), owing to allomorphy (e.g. *-zehn* (literally ‘ten’) as part of 13−19 in German, but -zig/-ßig as part of 30−90). (iii) Complex numerals built with forms representing 6−9 tend to be less canonical than those built with forms representing 1−5 (compare 11−15 with 16−19; 21−25 with 26−29; and 30−50 with 60−90). This is due to the quinary substructure present in several numeral systems in the sample, which are considered decimal in broader terms (cf. figure 2).

Finally, we zoom in to greater detail, and investigate whether we can find any patterns in the way the different transparency, canonicity and compositionality rules are satisfied across languages (figure 3). Figure 3a shows whether each transparency rule is satisfied in each decimal language of our sample. Numeral systems have been grouped by a hierarchical clustering algorithm based on the similarity of the set of rules satisfied by them, by minimizing the variance within groups (Ward method, see electronic supplementary material). This analysis shows six well defined clusters (figure 3a). It can be seen that most languages satisfy either all or none of the rules in the six different sets (i)–(vi), and this is reflected in the resulting clusters. For example, most languages in the first cluster from the right are almost perfectly transparent (all or most rules satisfied), most languages in the second cluster from the right satisfy all rules except those in set (v) (10 is not a part of 20, 30, …, 90), while languages in the first three groups from the left do not satisfy the rules in set (i) (10 is not a part of 11, 12, …, 19).

Figure 3b shows the outcome of canonicity rules either being satisfied or not. There is a much simpler structure, with only three clusters present. The rightmost cluster presents perfect canonicity; the middle cluster presents some languages featuring some of the most common irregularities, mostly regarding decades (e.g. Spanish 20 *veinte* as an atomic value rather than *dos-diez). In the left cluster, we again observe how complex numerals formed by 6−9 lose their perfect decimality owing to a quinary substructure (see figure 2b).

Finally, figure 3c, depicting compositionality, shows a much more regular pattern. The two clusters on the right are (almost) perfectly compositional, except for the number 20, which is atomic (not formed by 2 and 10) in the second cluster. The two clusters on the left present some other irregularities: the leftmost has some issues with the twenties, and the second from the left has several irregularities, mostly centred in the decades.

## 5. Discussion

We have analysed a genetically and geographically balanced sample of numeral systems regarded as decimal in languages of the world. First, although transparency patterns seem to show certain geographical structure, canonicity and compositionality patterns do not (figure 1). A possible explanation for this might be the areal nature of language contact (e.g. [24] for the case of numeral borrowing in India) affecting the forms for numerals rather than their underlying structure. Alternatively, the clustering of less transparent systems might correspond to regions that developed these systems early on, so that they have accrued layers of sound changes and other idiosyncratic changes that render them less clearly compositional.

Secondly, we see that rules involving lower numerals (atoms) are more often satisfied than those involving the base-10 (e.g. in the word for 17, it is more common to find the word for 7 than that for 10, i.e. 17 = *X* + 7 is more often satisfied than 17 = 10 + *Y*) in the transparency analysis, while the opposite is true for the canonicity analysis (figure 2a,b). This is due to the presence of allomorphs in the former case (different forms to express 10 in complex numerals, which do not necessarily coincide with the word for the numeral for 10, e.g. English 17 *seven-teen,* 70 *seven-ty*), and to the presence of lower anchors such as 5 in the latter case (if 7 = 5 + 2, and 17 = 10 + 5 + 2, then 7 is not represented as a solitary atom in 17). We can also notice these patterns when considering the aggregated transparency and canonicity scores for each numeral across languages (figure 4). The compositionality rules function as a control, remaining agnostic to distinctions between surface forms and base components.

Finally, the languages studied form clear clusters in terms of the rules satisfied by them in all three analyses, for transparency, canonicity and compositionality (figure 3). For the transparency study, we can identify six clusters, grouped mostly by which sets of rules (i)–(vi) are satisfied (see §3). For the transparency study, we identify three clusters, corresponding to the (almost) perfectly decimal systems, and those mixed with lower anchors, especially quinary (anchor-5). Finally, for the compositionality analysis, we observe four clusters, with all systems having very high scores on average.

Taken together, our findings demonstrate that decimal systems, while ubiquitous in distribution, exhibit considerable internal diversity in how numerical composition is realized. This diversity is not random: languages cluster into coherent types based on the structural rules they satisfy, with recurrent patterns of deviation that reflect deeper principles of language organization. Crucially, our threefold analysis reveals a typological asymmetry: even when transparency at the surface level is low, the underlying compositional and canonical structures tend to remain intact. This suggests that pressures for regularity in numeral formation operate not only at the level of phonological forms but also at more abstract representational levels. These insights have implications beyond numeral typology, contributing to broader debates about the interplay between form, function and learnability in linguistic systems. Future work could expand this approach to other base systems or explore the cognitive and historical processes that give rise to these structured deviations.

## Data Availability

All data and code can be accessed from [25]. Supplementary material is available online [26].

## Footnotes

^1^
In this paper, we are considering exclusively lexical numeral systems, i.e. the words used for representing numbers in languages of the world, independently of their notation or any other representation.

^2^
The word for 20, *zwan-zig*, can ultimately be analysed as ‘two ten’, making this an example of a decimal construction.

^3^
We are intentionally not calling these systems 'vigesimal' (base-20) and 'binary' (base-2), since these main compositional elements are often not used as proper bases, which would entail having special words for their powers (e.g. 400 = 20² and 4 = 2², respectively) [13].

^4^
Although a perfectly compositional system featuring a base different from 10 would share these advantages, there are two reasons why we are not considering them: (i) we want to focus on decimal systems, which are the only ones globally spread, across both geography and language genealogy, and (ii) non-decimal systems usually make use of at least two ‘anchors’ (numerals reused in order to form higher numerals; see a more strict definition in the following paragraph), such as 5 or 10 for numerals lower than 20 in vigesimal systems.

^5^
More precisely, this is the definition of ‘construction base’ or ‘anchor’. For details on the distinction between these two terms, see [13,21].

^6^
We are only considering deviations from perfect decimality. We could also define a broader typology, where we would consider whether the operator is overtly expressed (e.g. twenty-and-one versus twenty-one) or describe the different orders of the components (e.g. twenty-one versus one-twenty).

^7^
These are 37 numerals for each system, representing 1, 2, …, 29, 30, 40, 50, 60, 70, 80, 90 and 100.

^8^
Notice that, although 489 of the total set of 3898 languages with annotated bases are binary, making these systems the third most common (behind quinary and ahead of vigesimal), these systems tend to lack conventionalized forms for values beyond 4 or 5, therefore not reaching a ‘full set’ of numerals. In terms of [21], only some of them have proper cycle bases, while others merely use 2 as their main anchor.

^9^
Although subtraction and division (fractions) are sometimes involved in the formation of complex numerals in decimal systems, we are not considering them owing to their low frequency.

^10^
The word canonicity is in this context equivalent to perfect decimality. We use the former here in order to extrapolate it more easily to other bases.

^11^
Although not ultimately composed by two atomic numerals, we consider here the twenties (21–29) for the following reason: the twenties, as well as higher interdecadal numerals (31–39, 41–49, ..., 91–99), tend to show higher transparency scores than the teens (11–19). We use the twenties as a proxy for the interdecadal numerals in general. In addition, we gloss them as e.g. 27 = 20 + 7 rather than 27 = 2 × 10 + 7, since the internal structure of 20 is already accounted for in the sets of rules (v) and (vi).

^12^
The ligature *kɪ́* is ignored, since we are only interested in the presence of the forms for 5 and 2. Also, in the construction of the gloss ‘5 + 2’, we only considered structure and not perfect word form match (ràmːá is an allomorphic form of *ɾámːá*). If we were testing (quinary) transparency, the rule ‘Is the word for 2 included in the word for 7?' would not be satisfied.
